# Standardised Sampling Approach for Investigating Pathogens or Environmental Chemicals in Wild Game at Community Hunts

**DOI:** 10.3390/ani12070888

**Published:** 2022-03-31

**Authors:** Denny Maaz, Carl Gremse, Kaya C. Stollberg, Claudia Jäckel, Smita Sutrave, Carolyn Kästner, Birsen Korkmaz, Martin H. Richter, Niels Bandick, Julia Steinhoff-Wagner, Monika Lahrssen-Wiederholt, Anneluise Mader

**Affiliations:** 1 German Federal Institute for Risk Assessment (BfR), Max-Dohrn-Str. 8-10, 10589 Berlin, Germany; cmgremse@gmail.com (C.G.); kaya.stollberg@bfr.bund.de (K.C.S.); claudia.jaeckel@bfr.bund.de (C.J.); smita.sutrave@bfr.bund.de (S.S.); carolyn.kaestner@bfr.bund.de (C.K.); birsen.m.korkmaz@gmail.com (B.K.); martin.richter@bfr.bund.de (M.H.R.); niels.bandick@bfr.bund.de (N.B.); jsw@tum.de (J.S.-W.); anneluise.mader@bfr.bund.de (A.M.); 2 Federal Ministry of Food and Agriculture (BMEL), Wilhelmstraße 54, 10117 Berlin, Germany; monika.lahrssen-wiederholt@bmel.bund.de

**Keywords:** monitoring, zoonosis, contaminant, pathogen, hunting, game meat, sampling, wild boar, deer

## Abstract

**Simple Summary:**

Wild game can host pathogens capable of infecting humans, livestock, and companion animals or accumulate environmental chemicals that may be transferred via food of animal origin. For food safety research, as well as for various other scientific purposes, the provision of a sufficient number of samples without unnecessary disturbance or killing of animals is a major limitation. With the presented approach, it was possible to obtain samples from game animals killed as part of standard ungulate management practice. Examples of organs, tissues, and other matrices that have been used in wild ungulate studies in Europe and that may be obtained through this approach are summarised as well. The basis of this approach was a framework agreement with the BImA, whereby federal forest officials carried out sampling with the help of hunters at drive hunts from 2017/18 to 2020/21 in Brandenburg, Germany. Numerous samples from four ungulate species were obtained. The number of sampled animals per hunt differed between hunting districts and hunting seasons. Districts with higher hunting bags also promise higher sampling success. This approach can serve as the basis for long-term monitoring of animal and public health threats associated with wildlife and is adaptable to other regions.

**Abstract:**

Wildlife may host pathogens and chemicals of veterinary and public health relevance, as well as pathogens with significant economic relevance for domestic livestock. In conducting research on the occurrence and distribution of these agents in wildlife, a major challenge is the acquisition of a sufficient number of samples coupled with efficient use of manpower and time. The aim of this article is to present the methodology and output of a sampling approach for game animals, which was implemented from 2017/18 to 2020/21 at drive hunts in Brandenburg, Germany. The central element was a framework agreement with the BImA, whereby federal forest officials and other hunters collected most of the samples during field dressing. Further samples of game carcasses were obtained by scientists during subsequent gathering at a collection point. Altogether, 3185 samples from 938 wild ungulates of four species were obtained for various studies analysing—in this case—food-borne agents in game animals. Sampling was representative and reflected the proportional distribution of ungulate species hunted in Brandenburg. Hunting district and hunting season strongly influenced hunting bag and hence sampling success. This sampling approach was demonstrated to be a suitable basis for monitoring programs, that can be adapted to other regions.

## 1. Introduction

Knowledge about the distribution of zoonotic pathogens and contaminants of anthropogenic origin in wildlife is still limited, and continuous monitoring projects are rare, both in Germany and worldwide. In addition, wildlife disease monitoring for autochthonous and emerging diseases is an essential part of surveillance systems that are part of the One Health approach: public health, veterinary health, and ecological health [1].

Investigations of wild game by wildlife biologists, veterinarians, and public health professionals are usually limited in gaining access to wildlife samples in sufficient number and quality with a reasonable expenditure of time and manpower. Access over a defined period of years is often particularly important for investigating long-term effects. In contrast, those who work with wild game, such as foresters and professional hunters, generally have no capacities for scientific sampling. However, both groups are particularly interested in making use of hunted wildlife for research and/or monitoring purposes in order to avoid unnecessary killing and associated ethical challenges.

Monitoring projects on hunted wild game for the assessment of food safety risks often examine game meat or game meat products [2,3,4], as these are easily available as sample material. However, the predilection sites in the body for reliable detection of zoonotic pathogens or for measurement of levels of chemical contaminants are mostly located in organs and tissues that usually remain in the forest after field dressing or are disposed of in the course of game processing. For example, hepatitis E virus as well as levels of per- and polyfluoroalkyl substances (PFAS) are detected or determined predominantly in the liver [5,6], while gastrointestinal pathogens, such as *Cryptosporidium* spp., *Campylobacter* spp., or shigatoxigenic *Escherichia coli* (STEC) are predominantly in the faeces [7,8,9]. Serological evidence of an active or past infection by detection of antibodies is normally performed in blood serum or meat juice (e.g., [10,11,12,13]). In general, these sample matrices can only be obtained directly after the animals have been hunted.

In recent decades, a dramatic increase in the abundance of cervids and wild boar (*Sus scrofa*) in Europe has been observed [14,15,16,17]. Hunting of these animals is part of game management and is governed by federal and state hunting legislation in Germany. It is carried out by trained and licensed persons to avoid damage to agriculture and forestry, to reduce wildlife epidemics, to reduce vehicle collisions, and for game meat production. The yearly drive hunts from October to January are an essential part of these reduction measures for ungulate management. At that time of year, the hunting season is open for all ungulate species, the mating time is over, and the leafless forestry enables an unobstructed view for shooting. Drive hunts in Germany are usually organised with a varying number of hunters, distributed across the hunting area on fixed stands. A few beaters and trained dogs trawl noisily through the area and rouse the game so that it moves within an optimal shooting range of the hunters. These hunts last about three hours and usually take place in the morning until noon. Field dressing and gathering at a collection point for traditional presentation of killed game at the end of drive hunts are opportunities for obtaining samples from game animals.

A literature review of sampling techniques and parameters collected from selected peer-reviewed papers dealing with analysis of pathogens or contaminants in different organs, tissues, and body fluids in wild ungulates in Europe (*n* = 21), revealed that in the majority of studies, hunted animals were sampled, while in only two studies, faecal samples were taken from living game at feeding stations [7,18]. In five out of the 19 studies where only samples from carcasses were taken (meat, lymph nodes), game handling establishments were used in two of them [11,19], although this is likely also the case for the other three studies for which no details were given [20,21,22]. The remaining 14 studies [5,6,8,9,12,23,24,25,26,27,28,29,30,31] dealt with matrices that have to be sampled during field dressing (inner organs, serum, faeces). Two of them mentioned community hunts, and three studies stated that samples were provided by hunters. However, none of them provided further detail about the motivation of the hunters or how sampling was organised at hunts. In addition, thirteen studies sampled for one hunting season only, while seven sampled for two, and one for a period of three seasons. These results reveal that most studies omit a detailed description of how samples were obtained and that best practice examples for long-term sampling approaches in wild game are lacking.

The aim of the current paper is to present in detail the concept and the output of a standardised, large-scale, and long-term sampling approach for wild game that was conducted by the German Federal Institute for Risk Assessment (BfR) during four hunting seasons and is a feasible basis for research studies and monitoring of biological and chemical agents in wildlife.

## 2. Materials and Methods

The basis of the sampling approach was a framework agreement involving the German Federal Institute for Risk Assessment (BfR) and the German Institute for Federal Real Estate (BImA). The federal forest division of the BImA is responsible for forest and game management over 476,000 ha of hunting area in Germany [32] and is organised in 17 regional sections managing 254 forest districts [33]. In the four hunting seasons from 2017/18 to 2020/21 presented here, sampling was conducted in collaboration with two of these regional sections in the federal state of Brandenburg. The framework agreement was initiated since a previous project almost failed because the sampling was based on a voluntary basis. In addition, four drive hunts were visited in two hunting districts of the state forest of Brandenburg and one drive hunt on a private hunting ground in Brandenburg.

**Figure 1:**
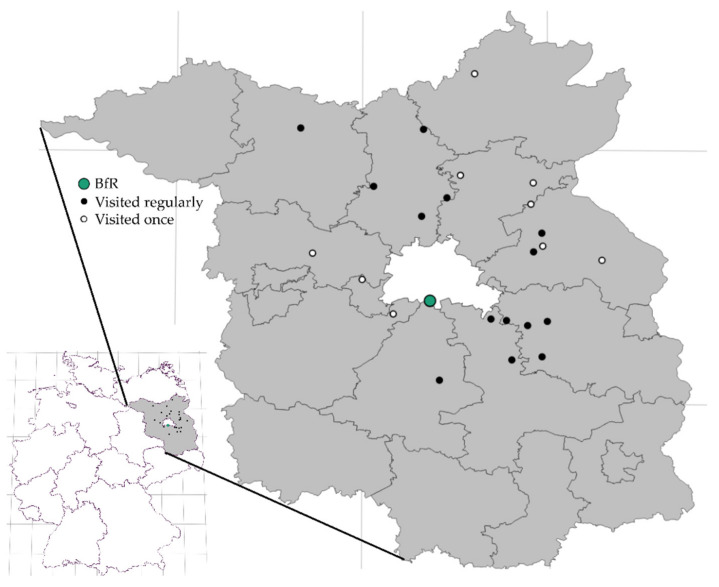
Geographic distribution of hunting districts in the German federal state of Brandenburg where game animals were sampled. The areas of Brandenburg and Berlin are seen in grey and white, respectively. The 13 administrative districts and 3 district-free cities of Brandenburg are bordered. The location of the BfR in Berlin is marked with a green circle. White districts were visited once and black districts regularly.

In early summer, dates for the drive hunts at the properties of both regional sections of the federal forest division from October to January were communicated to the BfR. This allowed for an early planning of necessary manpower and equipment for sampling, as well as laboratory capacities for analyses. In most hunting districts, drive hunts were organised twice in a hunting season. The drive hunts where sampling was carried out were selected in such a way that the hunting districts promise high numbers of game and imply less than two hours driving time from the institute (Figure 1).

A vehicle, ideally with four-wheel drive, was required for the entire drive hunt season, to transport equipment and sampling personnel to the forest hunts, which were usually only accessible through unpaved roads. Equipment included transport boxes with cold packs, knives, and personal protective clothing (high visibility vest and caps, gloves). A zip-lock PE plastic bag was prepared for every expected animal and filled with PE plastic bags, as well as 50 mL or 100 mL PP screw cap containers for every organ, tissue, faeces, or blood sample to be taken, together with a laminated, one-page easy-to-understand sampling instruction. A cordless outdoor cleaner, tissues, and hand sanitiser were used for cleaning of hands and equipment in the field. Two to four samplers from the BfR usually took part in every hunt.

The drive hunts usually started with a meeting of all participants at a central place in the forest at 7:30 a.m. During the initial briefing of the leader of the hunt, general instructions including safety rules, best hunting principles, time scheme, and open game, together with an explanation of how the samples should be taken for the BfR, were communicated. Then, hunters were grouped together with a group leader, who was usually a staff member of the forest division. The BfR samplers distributed prepared plastic bags with the sampling containers among the group leaders. Subsequently, hunters proceeded to their stands, and the drive hunt usually started at 8:30 a.m. and ended at 12:00 p.m. The samplers also took part as hunters or as beaters. Hence, they supported the hunt, which was highly welcome and increased the motivation of the hunters for sampling.

After the hunt, game shot within a group was controlled by the group leader and usually field dressed on-site by the hunters. In that case, hunters themselves took samples of heart, liver, spleen, blood (scooped from the chest cavity), faeces, nasal swabs, and caecum. Samples were then placed into the allocated sample containers according to the one-page instructions located in the sampling bag. The game identification number was written on the bag as the key identifier. Afterwards, game carcasses were transported by car to a collection point, where the gathering of the hunters took place. The hunters handed over the obtained samples, which were then either actively or passively cooled, depending on outside temperature. Further samples were obtained by BfR samplers directly from the collected game carcasses, such as tongue, tonsils, larynx, abdominal fat, and foreleg, abdominal, or diaphragm musculature. Therefore, a minimum of two samplers was required on such hunts. Less frequently, hunts were organised in such a way that all killed game was transported to the central point to be field dressed by the staff of the forest division. In this case, at least four persons were required to achieve a sufficient output, since samples had to be taken actively during simultaneous field dressing at two or more gutting racks.

At the end of each hunting day, a photograph was taken to document the list of killed game compiled by the leader of the hunt. This included data for game identification number, species, age class, sex, and carcass weight. The age classes for every ungulate species are defined in the Directive of the German federal states of Brandenburg and Mecklenburg-Western Pomerania [34]. They are divided into age classes 0 (juveniles, <1 year old), 1 (yearlings, ≥1 and <2 years), and 2 (adults, ≥2 years). Two additional age classes apply for male Cervinae: for red deer age classes 3 (≥5 and <10 years) and 4 (≥10 years), and for fallow deer age classes 3 (≥3 and <8) and 4 (≥8). Game age was determined by hunters mainly based on physical appearance and antler development, and in the case of uncertainty, ultimately through dentition pattern and tooth wear.

Samples were cooled and transported to the BfR where every game animal received a laboratory serial number, which was used as its unique identifier within the institute. Game samples were aliquoted for further analysis and stored at 4 °C or −20 °C depending on the preferences of the analytical method for the biological or chemical agent to be tested. Blood was centrifuged at 1600× *g* for 10 min at 4 °C to obtain a clarified serum pool. All samples were used for food safety research.

Only blood, heart, and faeces were sampled throughout all hunting seasons and from all ungulate species. Tongue, diaphragm, fat tissue, foreleg musculature, larynx, and tonsils were sampled only from wild boar. Fallow deer (*Dama dama*) were, except for one in 2018/19, only sampled from 2019/20 onwards.

From 2018/19 onwards, a laboratory information management system (LIMS) was utilised using the software winLIMS (Version 9.1.12.28760, Quality Systems International GmbH, Rodermark, Germany) as part of quality-assured work in accordance with ISO 17025 accreditation. It allows for management of information on the complete lifecycle of samples including sample metadata, laboratory analysis, and sample storage and can be accessed from every lab in the BfR. The central storage of data enables cross-lab analysis bringing together information from several biological and chemical agents analysed in the same game animals.

Statistics were performed with GraphPad Prism version 8.2.0. Pearson correlations were calculated for sampling success for the four ungulate species together with their proportions within the total hunting bag of Brandenburg and for number of sampled animals per hunt together with the total hunting bag of the district of the respective hunt in 2018/19. A binomial test was performed to test whether the sex ratio of sampled animals differed from 50%. One-way ANOVA with a post-hoc Tukey’s multiple comparisons test was performed for the analysis of sampled animals per hunt between the hunting seasons. The map of all sampling locations in Brandenburg (Figure 1) was created using the software Flourish Studio (https://app.flourish.studio, accessed on 30 January 2022).

## 3. Results

### 3.1. Performance of the Sampling Approach

Already at the initiation of the sampling approach during the first hunting season 2017/18, the procedure worked well. In the oral instructions before the hunt, hunters and drive hunt group leaders were informed about which samples of which game species were to be taken, which was followed by the majority of hunters. They sampled shot game during field dressing near the place of shooting and brought the samples to the central display area, where the killed game was presented ceremonially. Here, for the scientists, there was enough time between the rites to obtain further samples of the animals. Only those few drive hunts where all shot game was field dressed simultaneously at a central spot were challenging, since the samplers had to collect and label all samples in a short span of time.

The acceptance of the sampling approach by the hunters increased from year to year. Participation of the BfR fit smoothly into the hunting process, and hunters as well as employees of the federal forest division appreciated the knowledge transfer with food safety experts. Unfortunately, in response to the COVID-19 pandemic in combination with the outbreak of African swine fever (ASF) in the federal state of Brandenburg during the hunting season 2020/21, several methodological changes had to be made by the federal forest division during drive hunts. These changes drastically affected sampling: The cancellation of oral instructions at the beginning of the hunt and the obligation of reduction of contacts and maintenance of appropriate distance for all participants of the hunt limited the opportunity for distribution of sampling bags to the hunters. Hence, the possibility of instructing and motivating them for the sampling was eliminated. Also, the ceremonial presentation of killed game at the end of the hunt was cancelled for the purpose of reducing personal contacts, which ruled out sampling there as well. In addition, the increased efforts of the federal forest division employees in response to the two outbreaks, including the concurrent sampling of wild boar for the detection of ASF, also reduced the possibility of coordinating sampling for the BfR.

Apart from these challenges in 2020/21, the approach was successful in sampling numerous game animals per hunting season. A total of 3183 samples were obtained from 938 ungulates from four species. Depending on the hunting season, samples were obtained at between 11 and 19 drive hunts from between 6 and 14 different hunting districts, with a total of 62 hunts at 23 districts over all four hunting seasons (Table 1). In some districts, more than one drive hunt was visited in a season. The geographic distribution of the 23 hunting districts in Brandenburg is shown in Figure 1. They were distributed across 10 out of the 13 counties of Brandenburg. In general, the time schedule of suitable drive hunts with a high bag expectation and the available manpower were pivotal in the choice of hunts, and studies associated with the game sampling approach had an inferior focus on continuous sampling in the same hunting districts in consecutive years. Consequently, only two districts were visited in all four years, while 12 were visited in only 2 years, and 9 only once. However, sampling in the same districts in consecutive years is possible as part of a regular monitoring, since the drive hunts take place every year due to the continuing need for game management.

In 2017/18, 279 wild ungulates were sampled followed by 188 in 2018/19, 381 in 2019/20, and 90 in 2020/21 (Table 1). Also, numbers of obtained samples were high in the first three hunting seasons: 744 in 17/18, 681 in 18/19, and 1353 in 19/20. Only during hunting season 2020/21, a smaller number of samples (407) was obtained as a consequence of several limitations due to the COVID-19 pandemic and ASF outbreak.

**Figure 2:**
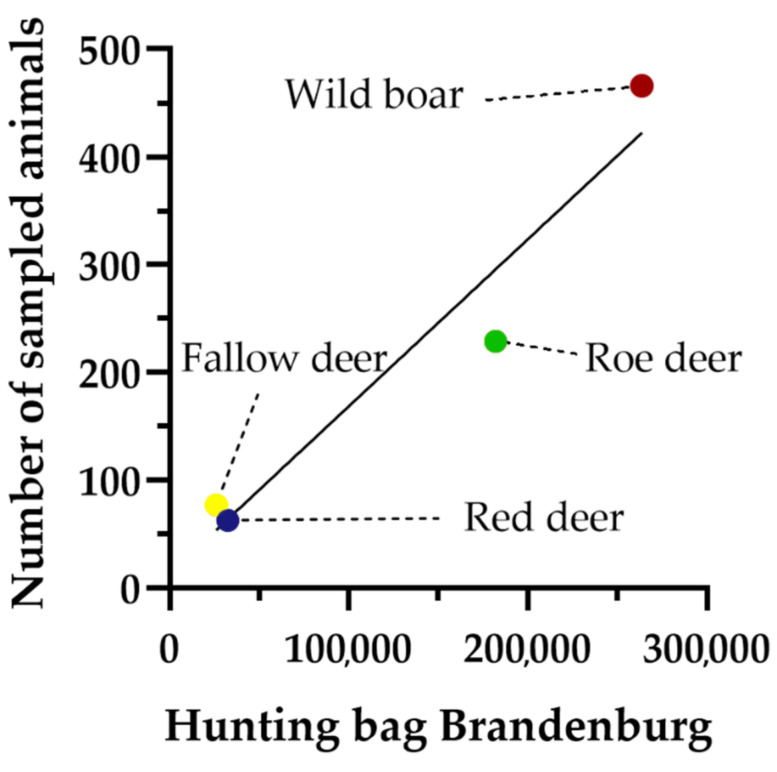
Number of sampled ungulates for all 4 species during the hunting seasons 2017/18, 2018/19, and 2019/20 in comparison to the total hunting bag for these species in Brandenburg during the same period of time. The diagonal line indicates a linear regression from the data revealing a strong positive correlation between sampled and hunted animals for the respective game species (R^2^ = 0.934, *p* = 0.033).

Wild boar was the most frequently sampled species with 488 individuals, followed by roe deer (*Capreolus capreolus*, 260), fallow deer (92), and red deer (*Cervus elaphus,* 85). Species identity of 13 ungulates remained unclear due to the lack of labelling of the sample bags. However, all of the unidentified animals were sampled in the first hunting season, before the methodology was optimised. It is certain that the proportional distribution of game species sampled with this approach was directly dependent on their proportion in the hunting bag of the drive hunts, which in turn is dependent on the local ungulate population. Figure 2 shows that relative sampling success for the four ungulate species in the hunting seasons 17/18, 18/19, and 19/20 strongly correlated positively with their proportions within the total hunting bag of Brandenburg during the same period of time (R^2^ = 0.93, *p* = 0.034) (data used: [35]). Slightly higher relative success of wild boar sampling occurred because nearly all carcasses of this species were sampled at the place of ceremonial presentation to obtain further tissues/organs for *Alaria alata* analysis [36]. Consequently, the mean number of samples obtained per animal, which was 3.4 for all species, was also slightly higher for wild boar.

**Figure 3:**
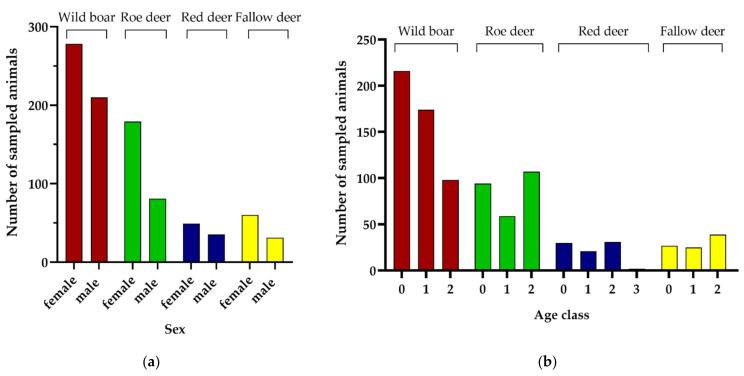
Distribution of sampled game animals across (**a**) sex and (**b**) age class for each of the four ungulate species.

Female animals were sampled more often than males (Figure 3a, *p* < 0.001), which was also significant for all hunting seasons and all species (all *p* < 0.006) except for red deer (*p* = 0.183). Particularly for roe deer and fallow deer, samples were obtained from approximately twice as many females as males. Distribution across age classes (Figure 3b) differed between species. While in wild boar, samples were less frequently obtained from the oldest animals of age class 2, relatively fewer animals of age class 1 were sampled in the three cervid species. Age class 3, which only applied for old male red deer, was rare.

Fourteen different organs/tissues were sampled during the presented four hunting seasons. The most frequently obtained samples were blood (668), faeces (631), heart (578), pooled samples of tongue, diaphragm, and abdominal fat (353), liver (340), spleen (210), and tonsils (151), followed by nasal swabs (75), abdominal muscles (69), foreleg musculature (58), larynx (35), and caecum (17). Apart from blood, faeces, and heart, differences between the number of organs/tissues arose mainly because they were only required in individual hunting seasons or only from individual species. However, particularly heart and liver are also used as dog treats by hunters and were accordingly less often obtainable for scientific purposes. In addition, the heart often showed major damage due to the bullet.

### 3.2. Parameters Affecting Sampling Performance

Although the methodology and sample demand for the associated studies changed slightly over the years, and some degree of the sampling success of a hunt had organisational causes (such as successful distribution of sampling bags among the hunters), some parameters having an additional influence became obvious.

**Figure 4:**
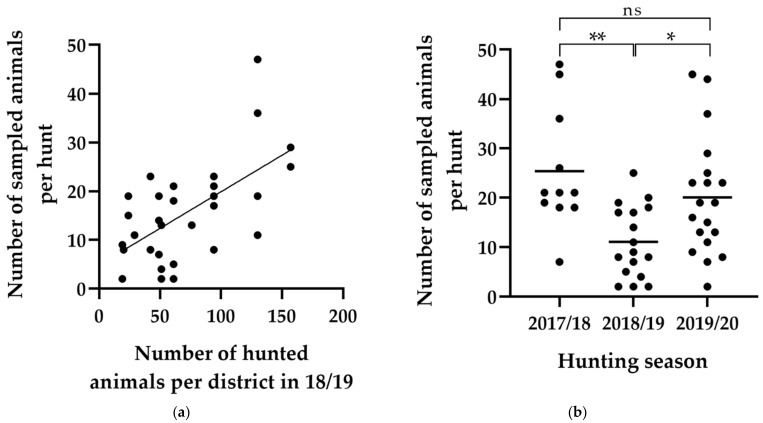
Number of sampled ungulates per hunt in the first three hunting seasons in relation to (**a**) the total number of hunted ungulates in the same hunting district in hunting season 2018/19 and (**b**) the season. The former only includes hunts from one of the two regional sections due to data availability. Each dot indicates one hunt. The diagonal line indicates a linear regression from the data (**a**) revealing a positive correlation between the number of sampled and hunted animals (R^2^ = 0.371, *p* < 0.001). Horizontal lines indicate the mean (**b**). ns, not significant; * *p* < 0.05, ** *p* < 0.01.

**Figure 5:**
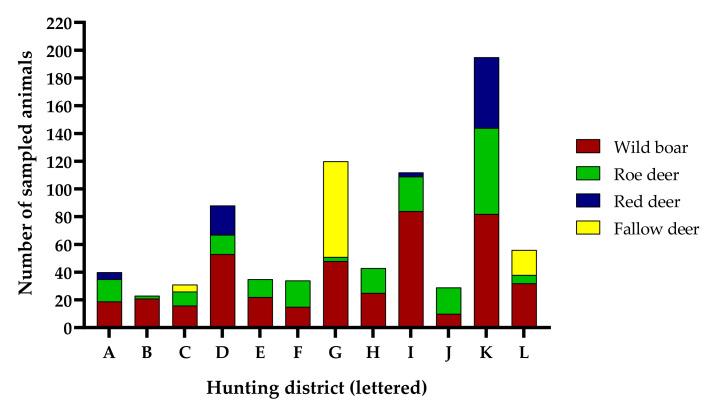
Number of sampled ungulates (all four hunting seasons) for all hunting districts where more than 20 animals were sampled.

The number of animals sampled per drive hunt ranged between 0 and 47, with a mean of 18.0 and median of 18 if season 2020/21 is excluded. The most obvious parameter affecting the sampling performance was found to be the hunting district. In districts where a high number of ungulates were hunted each year (data kindly provided by the BImA), sampling success was usually higher (Figure 4a). Also, the presence of a certain ungulate species was dependent on the hunting district (Figure 5). While roe deer and wild boar could be sampled at all of the 12 locations, with more than 20 animals sampled, red deer and fallow deer only occurred in four or three of these districts, respectively (Figure 5).

Sampling success also differed significantly between hunting seasons (Figure 4b, *p* = 0.003). In 2018/19 (*p* = 0.003 and *p* = 0.038, respectively) and 2020/21 (not shown), markedly fewer animals were sampled per hunt than in the other two seasons.

## 4. Discussion

A major challenge for research studies on wild game is the long-term acquisition of a sufficient number of samples. Most studies regarding the analysis of various biological and chemical agents in game do not describe in detail how sampling was organised, and best practice examples are lacking. The presented approach proved to be a representative and productive method for sampling game animals, which could be a basis for a continuous monitoring of wildlife zoonoses or contaminants. Thus far, over four hunting seasons, it was possible to obtain a wide variety of sample types from the four most common ungulate species in Germany, which represent the most important meat-producing game species [35]. A legally binding, long-term cooperation agreement between the two participating federal institutions for 10 years, enables a continuous use of districts and scheduled hunts as a basis for sample acquisition and monitoring.

The central element of the sampling approach was the commitment of federal forest officials and other hunters, who generally took the samples themselves following one-page instructions. Without their participation, the effort would require a high level of personnel expenditure for visiting all the shooting stands individually in order to sample the offal in addition to a greater disturbance of the hunting process due to the increased need for coordination. For the individual hunter, however, only a modest additional effort was required to take the samples of his/her killed game during evisceration. All BfR samplers were informed in advance about the process and practices of the hunt, and the BfR supported it when scientists attended training courses to obtain a hunting license. Hence, for federal forest officials, on the other hand, regardless of the framework contract, it was a benefit when experienced BfR employees supported the hunt and participated as either hunters themselves or beaters. Also, additional sampling of field-dressed game carcasses during ceremonial presentation, where BfR samplers obtained more difficult to extract tissues, such as tonsils, hardly interfered with the usual procedure. Hunters often expressed interest in the BfR studies and took this opportunity to inform themselves about them or about aspects of game meat safety in general. In addition, outside the drive hunt season, the results of these scientific studies were communicated through presentations by BfR scientists during information events for hunters, organised by the federal forest division on a yearly basis.

As shown, the distribution of sampled species reflects the distribution of the species in the hunting bag in Brandenburg. Hence, the sampling is representative at least in terms of the hunted species. While roe deer and wild boar are common throughout Central Europe, red deer and fallow deer only occur locally [37]. Accordingly, in contrast to roe deer and wild boar, they could only be sampled in certain hunting districts.

Unfortunately, no public data are available for Brandenburg for comparison on the distribution of the hunted game across age class and sex. However, there was no indication that the sampling showed a preference for a particular age class or sex. Female specimens were sampled more frequently than males in all four species. However, the comparatively more balanced sex ratio in wild boar may be due to the fact that sex can be hardly distinguished during hunting in age class 0, from which most samples were obtained. The main reason for the imbalance in terms of sex is probably the ungulate management plans that must be drawn up every year, at least for the cervid species. In Brandenburg, these plans are based on game management guidelines [34]. At least for the cervids included here, they specify a shooting ratio in the forest of 30:70 to 45:55 male to female specimens. Further specifications for the distribution of the kills over the age classes also roughly correspond to the ratio of sampled animals. There are no specifications for the sex ratio for wild boar. However, age specifications require that 80% of the bag should be animals in age classes 0 and 1, with age class 0 making up around two thirds. This proportion is also roughly reflected in the sampling.

As a result of these guidelines, the hunting bag only partially corresponds to the natural distribution of the species or their sex ratio and age groups. In addition, species with low or even declining populations, such as the European mouflon (*Ovis aries musimon*) in Brandenburg, are hunted infrequently or not at all. In the case of male cervids, especially adult red deer of age classes 3 and 4, high trophy prices (>€1000) also play a role in whether they are hunted or not. The bag also varies seasonally. The reason for this may be regulations regarding open and closed seasons, which are distinguished by species, sex, and age class, and are legally determined in Germany at both the federal [38] and state level [39]. Consequently, taking legal regulations into account, the obtained scientific results can only be seen as a representation of the hunted game population and not the complete wild game population. This should be emphasised during the presentation and publication of data.

Two parameters were identified that mainly affected sampling performance: the hunting district and the hunting year. Since sampling success was directly dependent on the hunting bag, districts with a higher annual bag also had a higher chance of a larger number of sampled animals. However, this has not always been the case, since the bag of a drive hunt may vary significantly within a year as well as between years. It mainly depends on the current distribution of the ungulate numbers in the forest district area at the beginning of the hunt and whether the surrounding districts have organised their own hunts on the same day. It also depends on the time of year, since at the beginning of the drive hunt season, wild boars in particular still predominantly reside in adjacent maize fields, while in winter, fallen snow usually increases the bag of roe deer, in particular due to better visibility. However, the hunting bag also depends on the number and quality of beaters, dogs, and hunters. A marked difference in sampling performance between the hunting years was not only observed in 2020/21 when the COVID-19 pandemic and the ASF outbreak disrupted sampling performance, but also in the first three years. This may be partly explained by the visitation of different forest districts or other methodological biases, such as a higher proportion of hunts where game was field dressed centrally, due to the omitted participation of the hunters in sampling. However, overall success in obtaining samples persisted throughout the first three hunting seasons and proves the applicability of our approach for regular monitoring.

The sampling approach was performed in drive hunts for ungulates, but can in principle also be used for other community hunts, e.g., for small game such as hares or water fowl. One limitation of this approach, however, is that drive hunts for ungulates are only organised in late autumn/winter, and hence samples can only be obtained at this time of year. This aspect must be taken into account, especially in studies on agents that display seasonality. Another limitation is the clustered distribution of game animals leading to large differences in hunting bag between hunting districts and, hence, a clustered sampling at sites with high game density. Depending on the focus, resulting studies may have to compensate for clustered, non-random sampling.

Basically, the availability of one sampler with a vehicle, a knife, plastic bags, and a box with cool packs is the prerequisite for the sampling, meaning that small research facilities are also capable of using the sampling approach. However, it is advisable to have more participants, because this way more samples in general and, in addition, more effortful samples can be obtained through distribution of tasks (documentation, sampling).

Access to wild game samples facilitated numerous studies at the BfR to close knowledge gaps addressing various food safety-relevant biological and chemical agents in the wildlife reservoir. A selection of different game organs and tissues that can be obtained with this approach and used for qualitative and quantitative analyses of diverse agents are listed in Table 2 and Table 3. However, the sampling approach may also be used to obtain samples for purposes beyond food safety, enabling collaborative, multisectoral, and transdisciplinary One Health approaches. Examples of such studies in the areas of public health, wildlife health, animal welfare, ecology, or phylogeography are listed in Table 4.

The analysis of different types of samples from the same animal, as well as the central sample distribution and data management simultaneously enabled a meaningful multiple use of the animal and reduced costs and effort for every study. It also enables a comprehensive view of consumers’ exposure to different food-borne risks from game meat. Furthermore, it is an ideal opportunity for investigating the occurrence of frequent co-infections in order to detect potential inter-relationships between the pathogens due to analogous life cycles, true interaction, or other causes.

The participation in the community hunts for sampling can also be used for other peripheral studies. Many hunters voluntarily participated on-site in surveys about different aspects of game meat production and safety as well as in practical evaluation of the quality of their shooting distance estimations. Also, peripheral experiments were possible, such as on game handling practices or microbial hygiene.

The presented approach is suitable for ensuring a standardised, large-scale, and long-term sampling of organs, tissues, and other matrices of wild game with relatively little effort and with an advantage for all parties involved. It thus overcomes typical field constraints and logistical or ethical challenges and enables research on various aspects of public health, wildlife diseases, and ecology.

## 5. Conclusions

The presented standardised sampling approach for wild game animals proved to be representative for hunted game and effective for obtaining a large number of samples of different organs, tissues, and other matrices. Essential prerequisites were a framework contract between scientific and game management institutions and thorough training in hunting for all participants. The sampling facilitated numerous studies on food safety at the BfR and will be adapted to reference areas throughout Germany. Hence, the approach is an applicable basis for long-term monitoring programs and One Health research in Europe at the interface of wildlife, public, and ecological health.

## Figures and Tables

**Table 1:** Number of locations and drive hunts (upper part) where samples were obtained, as well as number of game animals and obtained samples (lower part) in the first four hunting seasons. The last row (lower part) shows the total number of sampled animals/samples across all game species. The last column shows the total number of locations and hunts, as well as of all animals or samples obtained across all hunting seasons.

	Hunting Season									
	2017/18		2018/19		2019/20		2020/21		Total	
number of hunting districts	6		10		14		11		23	
number of drive hunts	11		17		19		15		62	
game species	animals	samples	animals	samples	animals	samples	animals	samples	animals	samples
red deer	41	114	23	51	13	45	8	31	85	241
fallow deer	0	0	1	1	62	275	29	142	92	418
roe deer	79	140	84	250	66	215	31	125	260	730
wild boar	146	456	80	379	240	818	22	109	488	1762
unknown ungulate	13	34	0	0	0	0	0	0	13	34
total	279	744	188	681	381	1353	90	407	938	3185

**Table 2:** Examples of organs, tissues, and other matrices of wild ungulates that have been analysed in different studies in Europe for detection of zoonotic pathogens. The asterisks indicate pathogens that were also analysed in the game samples at the German Federal Institute for Risk Assessment (BfR).

Organ/Tissue	Pathogen Investigated	Detection Method	Disease in Humans	Cases in Germany ^1^ 2001–2020	Source
Liver	Hepatitis E virus * (*Orthohepevirus* A)	RNA	Hepatitis E	32,144	[5]
Heart	*Toxoplasma gondii* *	DNA, Antibodies (host response)	Toxoplasmosis (congenital/ postnatal)	1438 (postnatal)	[13,24,40]
Blood/Serum	All pathogens	Antibodies (host response)	-	-	e.g., [10,11,12]
Faeces	*Cryptosporidium* spp. *	DNA, Oocysts	Cryptosporidiosis	29,867	[7,9,18]
	*Giardia* sp.	Cysts	Giardiasis	74,852	[9]
	*Campylobacter* sp. *	Culture	Campylobacteriosis	1,341,087	[8]
	Shigatoxigenic *Escherichia coli* (STEC) *	Culture	Haemolytic uremic syndrome (HUS) and bloody diarrhoea	2254 (HUS) ^2^ 39,665 (EHEC) ^3^	[8]
	*Salmonella* sp.	Culture	Salmonellosis	687,133	[8]
	*Rotavirus* sp. *	RNA	Rotaviral gastroenteritis	962,075	[25]
Spleen	*Brucella* sp. *	DNA	Brucellosis	745	[26]
Tonsils	*Yersinia* sp. *	DNA, Culture	Yersiniosis	87,337	[27,28]
Nasal swabs	Methicillin-resistant *Staphylococcus aureus* (MRSA) *	Culture	Various manifestations	35,936	[8]
Lymph nodes	*Mycobacterium* spp.	DNA, Culture	Tuberculosis	109,460	[22]
Tongue, Abdominal fat	Alaria alata *	Mesocercariae, DNA	-	-	[19,36]
Foreleg musculature, tongue, diaphragm	*Trichinella* sp.	First larvae	Trichinellosis	98	Regulation (EC) 2015/1375 ^4^

^1^ Source: SurvStat@RKI 2.0 https://survstat.rki.de (accessed on 22 February 2021); ^2^ HUS, Haemolytic uremic syndrome; ^3^ EHEC, Enterohaemorrhagic *Escherichia coli*; ^4^ Examination mandatory for wild boar intended for human consumption.

**Table 3:** Examples of organs or tissues of wild ungulates that have been analysed in different studies in Europe for detection of contaminants or residues. The asterisks indicate chemicals that were also analysed in the game samples at the German Federal Institute for Risk Assessment (BfR).

Organ/Tissue	Chemical Investigated	Source
Liver	Per- and polyfluoroalkyl substances (PFAS) *	[6]
	Pesticides (herbicides, insecticides, rodenticides) *	[20,29]
Kidney	Heavy metals (cadmium, mercury, lead, …)	[30]
Fat tissue	Dioxins and dioxin-like PCB	[4]
Musculature	Cesium-137	[21]

**Table 4:** Examples of organs, tissues, and other matrices of wild ungulates that have been analysed in different studies in Europe for reasons not associated with food safety.

Organ/Tissue	Investigated for	Source
Blood	Nutrition (essential elements), oxidative stress	[41]
	Haptoglobin (marker of inflammation and sub-clinical disease)	[42]
	African swine fever virus	[43]
Spleen	Phylogeography	[44]
Attached ticks	Ticks and host reservoir function for *Borrelia burgdorferi* sensu lato	[45]

## Data Availability

The data presented in this study are openly available in FigShare at https://doi.org/10.6084/m9.figshare.19383785.v2 (accessed on 18 March 2022).
